# Joint modeling of progression-free survival and patient-reported outcomes to evaluate the association between disease progression and symptoms among patients with relapsed/refractory multiple myeloma

**DOI:** 10.1186/s41687-025-00943-9

**Published:** 2025-10-14

**Authors:** Stefan Knop, Hermann Einsele, Devender Dhanda, Thomas S. Marshall, Laurie Eliason, Dylan McLoone, Clyde Caisip, Jenny M. H. Chen, Doris Boehm, Amol D. Dhamane, Karthik Ramasamy, Shannon Cope, Kevin Towle

**Affiliations:** 1 Department of Hematology and Oncology, Nuremberg General Hospital, Paracelsus Medical School, Nuremberg, Germany; 2 Department of Internal Medicine II, Würzburg University Hospital, Würzburg, Germany; 3 Bristol Myers Squibb, Lawrenceville, NJ USA; 4 Precision AQ, Vancouver, BC Canada; 5 Bristol Myers Squibb, Munich, Germany; 6 Oxford University & Oxford University Hospitals NHS Trust, Oxford, Oxfordshire UK

**Keywords:** Joint models, Patient-reported symptoms, Relapsed/refractory multiple myeloma, Health-related quality of life

## Abstract

**Background:**

Here we aim to evaluate the relationship between progression-free survival (PFS) and patient-reported symptoms (measured by health-related quality of life scores) among patients with relapsed/refractory multiple myeloma (RRMM).

**Methodology:**

Pain and fatigue were identified as the most common patient-relevant symptoms within RRMM based on a predefined literature review of patient preference/qualitative studies (confirmed by clinical experts). Consequently, the European Organisation for Research and Treatment of Cancer QLQ-C30 pain, QLQ-MY20 disease symptoms (pain in different locations), and QLQ-C30 fatigue domains were selected. Change from baseline scores per symptom domain was jointly modeled with PFS assuming a current slope association structure. For each symptom, we evaluated trial-specific joint models based on individual patient data from 7 RRMM clinical trials. The association between symptoms and PFS was summarized via association-effect hazard ratios (HRs) from the joint models, where a HR > 1 indicates that symptom worsening was associated with an increased hazard of a progression/death event. Meta-analyses were performed to synthesize the joint model HRs from all trials into one summary statistic (meta-HR) per symptom domain.

**Results:**

Across trials, worsening in pain and fatigue was associated with an increased hazard of progression events (disease progression/death) based on joint-model-association-effect HRs. Specifically, meta-HRs (95% CI) were 1.10 (1.02, 1.19) for QLQ-C30 pain, 1.10 (1.01, 1.20) for QLQ-MY20 disease symptoms, and 1.11 (1.05, 1.17) for QLQ-C30 fatigue.

**Conclusions:**

This study demonstrated that worsening in pain and fatigue was consistently associated with an increased hazard of disease progression or death events across numerous RRMM clinical trials with varying disease severity. This suggests risk of PFS events may align with patient experience in terms of worsening symptom burden.

**Supplementary Information:**

The online version contains supplementary material available at 10.1186/s41687-025-00943-9.

## Introduction

Increasing emphasis on the patient experience has led to an interest in considering patient-reported outcomes in combination with clinical outcomes (e.g., survival) when evaluating the effectiveness of new interventions [1–5]. For example, while progression-free survival (PFS) is a common primary endpoint in oncology studies, because of its established criteria based on laboratory diagnostics and imaging procedures, it has been critiqued for disregarding changes in patients’ perceived health-related quality of life (HRQoL) [6, 7]. Patient-reported symptom burden and HRQoL are important to understand the patient perspective and disease experience. While there is some evidence to suggest that the magnitude of PFS improvement depends on the severity of disease-related symptoms in oncology, there are mixed conclusions regarding the association between PFS and patient-reported outcomes, depending on the analysis methodology and the underlying variability in the cancer types and symptoms evaluated [8–12]. Therefore, additional research on the relationship between PFS and disease-specific symptoms is required to support decision-makers.

Clinical trials usually analyze outcomes separately via independent models, such as Cox proportional hazards models for time-to-event outcomes (i.e., PFS) and linear mixed-effect models for longitudinal data with repeated measures over time (i.e., patient-reported symptoms/HRQoL), for example. However, by ignoring potential correlations between outcomes, separate analyses may result in biased estimates in certain situations [13]. Alternatively, joint models allow for the simultaneous analysis of time-to-event outcomes (i.e., PFS) and longitudinal outcomes (i.e., HRQoL). In comparison with models evaluating each outcome separately, joint models have the benefit of accounting for correlation between outcomes, reducing bias, allowing for the inclusion of longitudinal covariates, increasing power, evaluating different clinical research questions, and permitting specification of coprimary outcomes of interest [13–15]. More specifically, joint models allow for the impact of symptoms/HRQoL on PFS to be quantified, which is important considering the increasing emphasis on the patient experience and requirements from the German Institute for Quality and Efficiency in Health Care (IQWiG) to consider both mortality/morbidity and symptoms or HRQoL during treatment benefit evaluations [16–19].

Consideration of a composite endpoint of PFS (disease progression or death events) in combination with a patient-relevant outcome was submitted to IQWiG as part of a benefit assessment of daratumumab in relapsed/refractory multiple myeloma (RRMM) based on the APOLLO trial. While IQWiG identified that “[s]ymptomatic disease progression is generally patient-relevant” (translated to English), it was noted that additional research would be needed to assess the association between disease progression and symptoms in RRMM, including the clear selection of relevant symptoms and transparent methods to assess the association [20]. To address this critique, the relationship between PFS and patient-reported symptoms was assessed (as measured by validated HRQoL questionnaires) by using data from 7 RRMM clinical trials with different underlying patient populations in terms of disease severity and prior treatments.

## Methods

**Fig. 1:**
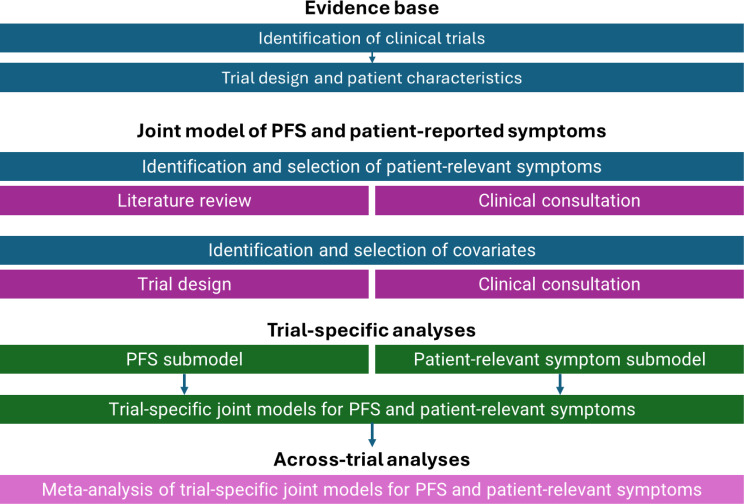
Summary of methodology. *PFS* progression-free survival

The following steps were performed to support this research question, and are summarized in Fig. 1: (1) clinical trials were selected based on eligibility criteria, and this evidence base was then summarized; (2) a literature review was performed to identify symptoms that were relevant to patients with RRMM; (3) clinicians were consulted to identify symptoms potentially related to disease progression and the most plausible model assumptions regarding the relationship; (4) models were fit to analyze PFS and symptoms separately (i.e., outcomes modeled independently) and then simultaneously (i.e., using joint models) for each trial and symptom domain; and (5) meta-analyses were performed to synthesize outputs from trial-specific joint models into one summary statistic for each symptom domain.

### Evidence base

#### Identification and selection of clinical trials

This study included any Bristol Myers Squibb (BMS) clinical trials (randomized controlled trials [RCTs] and single-arm trials) with available follow-up (i.e., completed primary analysis) evaluating the effectiveness of interventions for the treatment of RRMM with individual patient data (IPD) available for both PFS and HRQoL (as measured by the European Organisation for Research and Treatment of Cancer [EORTC] Core Quality of Life [QLQ-C30] and Multiple Myeloma Module [QLQ-MY20] questionnaires). The trials included OPTIMISMM [21], ELOQUENT-2 [22], KarMMa-3 [23], MM-003 [24], CC-92480-MM-001 [25], KarMMa [26], and CC-220-MM-001 [27].

#### Trial design and patient characteristics

The 7 trials were summarized in terms of study design characteristics, eligibility criteria, patient characteristics, outcome definitions, analysis of relative treatment effects, and handling of intercurrent events.

### Joint models of PFS and patient-reported symptoms

#### Identification and selection of patient-relevant symptoms

A literature review was conducted to identify which symptoms were most relevant to patients with RRMM to help inform the selection of domains from the EORTC QLQ-C30 and QLQ-MY20 questionnaires. These 2 questionnaires were selected because they have been validated as HRQoL instruments, have been accepted by health technology assessment (HTA) agencies, have specificity in terms of symptoms for patients with cancer (QLQ-C30) and patients with multiple myeloma (QLQ-MY20), and were prospectively collected in the trials [28, 29]. Predefined study selection criteria were used to identify patient preference or qualitative studies exploring patient symptoms/experiences in RRMM (unrestricted by interventions or comparators). The selection of patient-reported symptom domains was supported by clinical consultations with multiple myeloma experts (authors SK, HE, and KR; *N* = 2, Germany; *N* = 1, UK).

#### Identification and selection of covariates for trial-specific submodels

Trial-specific covariates were predefined for the PFS and symptom submodels and joint models based on trial design factors and clinical consultation. For the RCTs, trial-specific stratification factors were included as covariates, including age, number of prior treatments, high-risk cytogenetics, prior agents or status, and beta-2-microglobulin, as well as a covariate for the treatment effect. Clinicians recommended including the number of prior treatment lines per year as an indicator of disease aggressiveness for patients with RRMM; however, only KarMMa-3 and KarMMa reported the number of prior lines per year as a combined data source. This one variable was selected as a covariate for the KarMMa models, while for other trials, the number of prior treatments and time since diagnosis were both included as separate covariates as a proxy measure (including KarMMa-3, given that the number of prior lines was a stratification factor). Baseline HRQoL score and age were included as covariates for all trials for consistency. A full listing of covariates for trial-specific models is presented in Supplementary Table S1.

#### Trial-specific submodels and joint models for PFS and patient-relevant symptoms

The PFS submodel was a Cox proportional hazards model, fit separately for each trial. The PFS submodels assumed that the ratio of hazards was constant over time and that censoring was uninformed. Outputs were hazard ratios (HRs) with 95% confidence intervals (CIs), where HR > 1 indicated an increased hazard of a progression event (disease progression or death).

The trial-specific HRQoL-evaluable populations (patients who completed HRQoL questionnaires at baseline and at least one post-baseline assessment) were used for all models. The patient-reported symptoms submodel consisted of a linear mixed-effect model based on change from baseline for each symptom domain (separate models for each trial and symptom domain). Generalized linear mixed models were used to estimate the temporal trajectory of change from baseline, assuming the residual errors followed a normal distribution. All submodels included a random slope for the prognostic effect of time on symptoms. A random intercept term was also required for model convergence in OPTIMISMM to capture the between-subject variation. Lastly, it was assumed that symptom values were missing at random. Outputs were coefficients and standard error, where a positive coefficient value indicated that symptoms worsened with an increase in the respective covariate.

Joint models were fit for each clinical trial and symptom domain separately. Each joint model involved 2 submodels: (1) a Cox proportional hazards model for PFS, and (2) a linear mixed-effect model for change from baseline in symptom scores. The submodels were linked in the joint model using alternative association structures to characterize the risk of disease progression at a timepoint: current slope (risk depends on the slope of the symptom trajectory) or current value (the risk depends on the symptom value). The current slope association structure was selected, as clinicians believed that the rate of deterioration in symptoms was the more dynamic measure with predictive value. Current value association was selected as sensitivity analysis, which clinicians considered may be relevant for patients with aggressive disease that may progress sooner. Outputs from the joint model included: (1) coefficients and HRs (95% CIs) based on PFS and symptom jointly; and (2) association-effect HR (95% CI). For interpretation, an association-effect HR > 1 indicated that an increase in the slope of change from baseline symptom score (i.e., worsening of the symptom) corresponded to an increase in the hazard of a progression event (disease progression or death). Since OPTIMISMM was the only trial with time defined on a scale of years to allow for model convergence, the OPTIMISMM joint-model-association-effect HR was rescaled to months for consistency across the trials.

#### Meta-analysis of trial-specific joint models for PFS and patient-relevant symptoms

Meta-analyses were performed to synthesize the trial-specific joint model HRs into one summary statistic (i.e., a meta-HR of the association-effect HRs) for each symptom of interest. This meta-HR had the same interpretation as the association-effect HRs (i.e., meta-HR > 1 indicated that symptom worsening was associated with an increase in the hazard of disease progression or death). Random-effects meta-analyses were performed separately for each symptom using the log HRs and standard error of the log HRs from the 7 trial-specific joint models. Heterogeneity was evaluated using the I^2^ statistic.

#### Software

The PFS and symptom submodels were analyzed in R version 4.1.3 (http://www.r-project.org/) using the “survival” and “nlme” packages, respectively. The joint models were fit in a frequentist framework using the “JM” package in R. The meta-analysis was performed using the “metafor” package in R.

## Results

### Evidence base

#### Identification of clinical trials

Seven clinical trials with IPD that had completed primary analysis of PFS and HRQoL were available: OPTIMISMM [21] (evaluating pomalidomide + bortezomib + dexamethasone [PVd] vs. bortezomib + dexamethasone [Vd]); ELOQUENT-2 [22] (elotuzumab + lenalidomide + dexamethasone [ERd] vs. lenalidomide + dexamethasone [Rd]); KarMMa-3 [23] (idecabtagene vicleucel [ide-cel] vs. standard of care [SoC]); MM-003 [24] (high-dose dexamethasone [HiDex] vs. pomalidomide [POM] + low-dose dexamethasone [LoDex]); CC-92480-MM-001 [25] (mezigdomide + dexamethasone); KarMMa [26] (evaluating ide-cel); and CC-220-MM-001 [27] (iberdomide + dexamethasone). These trials represented various treatment classes, including immunomodulatory agents, proteasome inhibitors, monoclonal antibodies, chimeric antigen receptor (CAR) T-cell therapy, and cereblon E3 ligase modulators.

#### Trial design and patient characteristics

Among the 7 included trials, 4 were RCTs (OPTIMISMM, ELOQUENT-2, KarMMa-3, and MM-003) and 3 were single-arm trials (CC-92480-MM-001, KarMMa, and CC-220-MM-001) (Table 1). All were open-label, multicenter trials conducted across global sites. MM-003, ELOQUENT-2, and OPTIMISMM were earlier trials (study start dates from 2011 to 2013), while CC-220-MM-001, KarMMa, CC-92480-MM-001, and KarMMa-3 were more recent (study start dates from 2016 to 2019).

**Table 1:** Summary of evidence base [21–27]

Characteristic	OPTIMISMM [21]	ELOQUENT-2 [22]	KarMMa-3 [23]	MM-003 [24]	CC-92480-MM-001 [25]	KarMMa [26]	CC-220-MM-001 [27]
ClinicalTrials.gov Identifier	NCT01734928	NCT01239797	NCT03651128	NCT01311687	NCT03374085	NCT03361748	NCT02773030
Trial design	Phase 3 RCT (open-label)	Phase 3 RCT (open-label)	Phase 3 RCT (open-label)	Phase 3 RCT (open-label)	Phase 1/2 single-arm (open-label)	Phase 2 single-arm (open-label)	Phase 1b/2a multicohort single-arm (open-label)
Interventions (full trial N)	PVd (*N* = 281) or Vd (*N* = 278)	ERd (*N* = 321) or Rd (*N* = 325)	ide-cel (*N* = 254) or SoC (*N* = 132)	POM + LoDex (*N* = 302) or HiDex (*N* = 153)	Mezi + Dex (*N* = 101)	ide-cel (*N* = 128)	Iber + Dex (*N* = 107)
Study start date	January 7, 2013	June 20, 2011	April 16, 2019	March 11, 2011	February 6, 2018	December 13, 2017	October 14, 2016
Age, years	≥ 18	≥ 18	≥ 18	≥ 18	≥ 18	≥ 18	≥ 18
Performance score	ECOG 0–2	ECOG 0–2	ECOG 0–1	ECOG 0–2	ECOG 0–2	ECOG 0–1	ECOG 0–2
Exposure to prior regimens	1–3 lines of therapy, including at least 2 consecutive cycles of a lenalidomide-containing regimen	1–3 lines of therapy	2–4 lines of therapy, including an immunomodulatory agent, a PI, and an anti-CD38 monoclonal antibody	≥ 2 lines of therapy, including at least 2 consecutive cycles of a lenalidomide-and-bortezomib-containing regimen	≥ 3 lines of therapy, including lenalidomide, pomalidomide, a PI, and an anti-CD38 monoclonal antibody	≥ 3 lines of therapy, including an immunomodulatory agent, a PI, and an anti-CD38 monoclonal antibody	≥ 3 lines of therapy, including an immunomodulatory agent, a PI, and an anti-CD38 monoclonal antibody
Refractoriness to prior regimens	Refractory to previous therapy; exclusion: disease progression during therapy or within 60 days of the last dose of a bortezomib-containing therapy	Refractory to previous therapy; exclusion: refractory to lenalidomide	Refractory to the last line of therapy	Refractory to bortezomib or lenalidomide, and the last line of therapy	Refractory to an immunomodulatory agent, a PI, an anti-CD38 antibody, and the last line of therapy	Refractory to the last line of therapy	Refractory to an immunomodulatory agent, a PI, an anti-CD38 antibody, and last line of therapy
Crossover	Not permitted	Not permitted	Crossover permitted	Crossover permitted	Not applicable (single-arm)	Not applicable (single-arm)	Not applicable (single-arm)
PFS criteria	FDA censoring (IRAC, IMWG)	FDA censoring (IRC, EBMT)	FDA censoring (IRC, IMWG)	EMA censoring (IA, IMWG)	FDA censoring (IA, IMWG)	FDA censoring (IRC, IMWG)	FDA censoring (IRAC, IMWG)
Timing of response evaluations	Every 21 days (+/−3 days) and time of discontinuation	Every 4 weeks (+/−1 week)	Every 28 days (+/−3 days) for Months 1–25, every 3 months for Months > 25	Every 28 days (+/−3 days)	Every 28 days (+/−2 days) and end of treatment	Monthly evaluations (+/−3 days) for the first 6 months and then every 3 months (+/−14 days)	Every 28 days (+/−2 days) and end of treatment
PRO questionnaire schedule of assessment	Day 1 of each 21-day treatment cycle (before treatment administration) and at the end-of-treatment visit	At baseline (prior to randomization), on Day 1 of each 4-week treatment cycle (28-day cycle), and at the end of treatment or study withdrawal	Screening, baseline, Day 1, monthly between Months 1 and 24, and every 3 months from 25 months	At baseline, on Day 1 of each 28-day treatment cycle, and at treatment discontinuation	Day 1 of each 28-day cycle (Cycle 1 Day 1 as baseline), as well as the end-of-treatment visit	Screening, baseline, Day 1, monthly between Months 1 and 6, and every 3 months up to 24 months or study completion	Day 1 of each 28-day cycle (Cycle 1 Day 1 as baseline), as well as the end-of-treatment visit

*Dex* dexamethasone, *EBMT* European Society for Blood and Marrow Transplantation, *ECOG* Eastern Cooperative Oncology Group, *ERd* elotuzumab + lenalidomide + dexamethasone, *EMA* European Medicines Agency, *FDA* Food and Drug Administration, *HiDex* high-dose dexamethasone, *IA* investigator-assessed, *Iber* iberdomide, *ide-cel* idecabtagene vicleucel, *IMWG* International Myeloma Working Group, *IRAC* Independent Response Adjudication Committee, *IRC* Independent Response Committee, *Rd* lenalidomide + dexamethasone, *LoDex* low-dose dexamethasone, *Mezi* mezigdomide, *N* sample size, *PFS* progression-free survival, *PI* proteasome inhibitor, *POM* pomalidomide, *PRO* patient-reported outcome, *PVd* pomalidomide + bortezomib + dexamethasone, *RCT* randomized controlled trial, *SoC* standard of care, *Vd* bortezomib + dexamethasone

All trials included adult patients (≥ 18 years of age) with Eastern Cooperative Oncology Group (ECOG) performance scores of 0–2, except KarMMa and KarMMa-3, which included patients with ECOG performance scores of 0 and 1. Differences in eligibility criteria regarding prior lines of therapy were observed across trials, with requirements of 1–3 prior lines in OPTIMISMM and ELOQUENT-2; 2–4 prior lines in KarMMa-3; ≥2 prior lines in MM-003; and ≥ 3 prior lines in CC-92480-MM-001, KarMMa, and CC-220-MM-001. KarMMa-3, CC-92480-MM-001, KarMMa, and CC-220-MM-001 also required patients to be triple-class exposed (i.e., previously treated with an immunomodulatory agent, a proteasome inhibitor, and an anti-CD38 antibody). All trials required patients to have progressive disease on or within 60 days of the last line of therapy. MM-003, KarMMa-3, CC-92480-MM-001, KarMMa, and CC-220-MM-001 required patients to be refractory to the last line of therapy; MM-003 further required patients to be refractory to lenalidomide or bortezomib; and CC-92480-MM-001 and CC-220-MM-001 further required patients to be triple-class refractory (to an immunomodulatory agent, a proteasome inhibitor, and an anti-CD38 antibody).

Key patient characteristics across the evaluated trials are summarized in Table 2. The trials are organized by increasing number of median prior lines, from 2 prior lines (OPTIMISMM and ELOQUENT-2) to 6 prior lines (KarMMa and CC-220-MM-001). The sample sizes of the analysis populations ranged from 74 (CC-92480-MM-001) to 603 (ELOQUENT-2). Median age was generally similar across the trials, with a range of 60 (KarMMa) to 68 years (OPTIMISMM), as was the distribution of sex (range of 52.7% [CC-92480-MM-001] to 60.8% males [KarMMa-3]). Across the trials, KarMMa-3 had the lowest proportion of patients with stage III disease (13.2%), while MM-003 had the highest (31.7%). There was also variation in the median time since diagnosis across the trials, ranging from 3.5 (ELOQUENT-2) to 7.1 years (CC-220-MM-001). The number of prior lines per year was only reported for KarMMa and KarMMa-3 but was derived for other trials to compare patient populations ([median number of prior lines]/[time since diagnosis]), with a range of 0.4 lines/year (OPTIMISMM) to 0.9 lines/year (MM-003 and KarMMa).

**Table 2:** Summary of patient characteristics among the trial-specific joint model analysis populations [21–27]

		OPTIMISMM [21]			ELOQUENT-2 [22]			KarMMa-3 [23]			MM-003 [24]			CC-92480-MM-001 [25]	KarMMa [26]	CC-220-MM-001 [27]
		PVd	Vd	Total	ERd	Rd	Total	Ide-cel	SoC	Total	POM + LoDex	HiDex	Total	Mezi + Dex	Ide-cel	Iber + Dex
N	--	240	209	449	298	305	603	211	108	319	279	138	417	74	126	79
Age	Median (range), years	68 (29–87)	68 (27–89)	68 (27–89)	67 (37–88)	66 (38–91)	66 (37–91)	63 (30–79)	63 (44–83)	63 (30–83)	64 (35–84)	65 (35–87)	64 (35–87)	67 (42–85)	60 (33–78)	62 (44–82)
	≤ 75 years	202 (84.2%)	176 (84.2%)	378 (84.2%)	251 (84.2%)	259 (84.9%)	510 (84.6%)	207 (98.1%)	100 (92.6%)	307 (96.2%)	256 (91.8%)	127 (92.0%)	383 (91.8%)	63 (85.1%)	123 (97.6%)	72 (91.1%)
	> 75 years	38 (15.8%)	33 (15.8%)	71 (15.8%)	47 (15.8%)	46 (15.1%)	93 (15.4%)	4 (1.9%)	8 (7.4%)	12 (3.8%)	23 (8.2%)	11 (8.0%)	34 (8.2%)	11 (14.9%)	3 (2.4%)	7 (8.9%)
Sex	Male	135 (56.2%)	108 (51.7%)	243 (54.1%)	176 (59.1%)	182 (59.7%)	358 (59.4%)	129 (61.1%)	65 (60.2%)	194 (60.8%)	165 (59.1%)	78 (56.5%)	243 (58.3%)	39 (52.7%)	75 (59.5%)	45 (57.0%)
ISS stage	I	128 (53.3%)	109 (52.2%)	237 (52.8%)	135 (45.3%)	133 (43.6%)	268 (44.4%)	129 (61.1%)	72 (66.7%)	201 (63.0%)	76 (27.2%)	33 (23.9%)	109 (26.1%)	29 (39.2%)	47 (37.3%)	36 (45.6%)
	II	74 (30.8%)	67 (32.1%)	141 (31.4%)	92 (30.9%)	96 (31.5%)	188 (31.2%)	54 (25.6%)	22 (20.4%)	76 (23.8%)	105 (37.6%)	50 (36.2%)	155 (37.2%)	31 (41.9%)	49 (38.9%)	31 (39.2%)
	III	38 (15.8%)	33 (15.8%)	71 (15.8%)	61 (20.5%)	64 (21.0%)	125 (20.7%)	28 (13.3%)	14 (13.0%)	42 (13.2%)	84 (30.1%)	48 (34.8%)	132 (31.7%)	14 (18.9%)	30 (23.8%)	12 (15.2%)
	Missing	0 (0%)	0 (0%)	0 (0%)	10 (3.4%)	12 (3.9%)	22 (3.6%)	0 (0%)	0 (0%)	0 (0%)	14 (5.0%)	7 (5.1%)	21 (5.0%)	0 (0%)	0 (0%)	0 (0%)
Number of prior lines	Median (range)	2 (1–3)	2 (1–3)	2 (1–3)	2 (1–3)	1 (1–4)	2 (1–4)	3 (2–4)	3 (2–4)	3 (2–4)	5 (2–14)	5 (2–17)	5 (2–17)	5 (3–13)	6 (3–16)	6 (3–23)
Time since diagnosis	Median (range), years	4.0 (0.2–25.9)	4.6 (0.4–21.8)	4.2 (0.2–25.9)	3.5 (0.3–17.3)	3.5 (0.1–16.2)	3.5 (0.1–17.3)	4.3 (0.6–21.8)	4.1 (0.7–17.7)	4.3 (0.6–21.8)	5.3 (0.6–30.0)	5.9 (0.9–21.1)	5.7 (0.6–30.0)	7.0 (1.1–26.8)	6.0 (1.0–17.9)	7.1 (1.8–24.5)
Number of lines per year	Median (range)	0.4 (0.1–5.0) ^a^	0.4 (0.1–2.5) ^a^	0.4 (0.1–5.0) ^a^	0.5 (0.1–9.9) ^a^	0.4 (0.1–26.1) ^a^	0.5 (0.1–26.1) ^a^	0.7 (0.1–5.2)	0.6 (0.2–2.9)	0.7 (0.1–5.2)	0.9 (0.2–4.5) ^a^	0.8 (0.1–2.8) ^a^	0.9 (0.1–4.5) ^a^	0.8 (0.2–2.7) ^a^	0.9 (0.4–3.8)	0.8 (0.3–2.3) ^a^
Baseline Β2M	Median (range), mg/L	3.3 (0.9–15.9)	3.3 (1.1–16.7)	3.3 (0.9–16.7)	––	––	––	3.0 (1.4–26.3)	2.8 (1.4–15.3)	2.9 (1.4–26.3)	4.5 (1.6–19.0)	4.4 (1.6–30.0)	4.5 (1.6–30.0)	––	3.8 (1.3–32.0)	––

Notes: Unless specified, values represent n (%). ^a^Calculated value (number of prior lines/time since diagnosis). *Β2M* beta-2-microglobulin, *Dex* dexamethasone, *ERd* elotuzumab + lenalidomide + dexamethasone, *HiDex* high-dose dexamethasone, *Iber* iberdomide, *ide-cel* idecabtagene vicleucel, *ISS* International Staging System, *Rd* lenalidomide + dexamethasone, *LoDex* low-dose dexamethasone, *Mezi* mezigdomide, *POM* pomalidomide, *PVd* pomalidomide + bortezomib, + dexamethasone, *SoC* standard of care, *Vd* bortezomib + dexamethasone

The definition of PFS was the same within RCTs (time from randomization to disease progression or death, whichever occurred first) and within single-arm trials (time from infusion/treatment to disease progression or death, whichever occurred first). OPTIMISMM, KarMMa-3, MM-003, CC-92480-MM-001, KarMMa, and CC-220-MM-001 used International Myeloma Working Group (IMWG) criteria for response/disease progression criteria, while ELOQUENT-2 used European Society for Blood and Marrow Transplantation (EBMT) criteria. All trials used an independent review committee for response assessments, except MM-003 and CC-92480-MM-001, which only used investigator assessments.

In accordance with guidelines from the QLQ scoring manuals, all trials defined adherence as the completion of ≥ 50% of items on the QLQ-C30 or QLQ-MY20 questionnaires. If adherence was violated, then HRQoL data were considered missing at the evaluated timepoint. Missing values were not imputed for primary analyses in any trials, except for ELOQUENT-2, where values were imputed for missing items using the average of present items for domains in which the participant responded to ≥ 50% of the items. While all trials evaluated HRQoL at baseline, the frequency of subsequent evaluations varied across the trials. OPTIMISIMM evaluated HRQoL at each 21-day cycle; ELOQUENT-2, KarMMa-3, MM-003, CC-92480-MM-001, and CC-220-MM-001 evaluated HRQoL at each 28-day cycle (switching to every 3 months at Month 25 for KarMMa-3); and KarMMa evaluated HRQoL monthly between Months 1 to 6 and then every 3 months until Month 24 or study completion.

### Association between PFS and patient-relevant symptoms based on joint models

A total of 18 patient preference/qualitative studies were identified, of which 12 reported specific information on self-reported symptoms. Pain (*N* = 12 studies [100%]) and fatigue (*N* = 9 studies [75%]) were the most common symptoms of concern identified among the evaluated studies. Consequently, 3 EORTC-defined patient-reported symptom domains were selected for analysis: (1) EORTC QLQ-C30 pain (2 questions; scale of 1–4 for responses); (2) QLQ-MY20 disease symptoms (assessment of pain in different locations; 6 questions; scale of 1–4 for responses); and (3) QLQ-C30 fatigue (3 questions; scale of 1–4 for responses) (see questions and scoring in the Supplementary File) [30]. The clinicians agreed that pain and fatigue were the most relevant patient-reported symptoms related to disease progression rather than treatment-related effects, noting that pain (particularly bone pain) was the most relevant symptom related to disease progression. Additionally, the clinicians noted that other domains in the questionnaires were less specific (e.g., functional scales assessing various activities that were not symptom-specific), may overlap with other factors (e.g., infection), or were more likely to be related to treatment side effects (e.g., constipation and diarrhea) for patients receiving retreatment.

### Trial-specific submodels and joint models for PFS and patient-relevant symptoms

Table 3 summarizes PFS HRs from the RCTs for PFS modeled alone (i.e., from the submodel) vs. PFS modeled with symptoms (i.e., from the joint model). Across all trials, joint models resulted in slight shifts away from the null effect (HR = 1), with narrower CIs in comparison to the models with PFS alone (i.e., greater precision in estimated treatment effects when patient-reported symptoms were incorporated with PFS).

**Table 3:** Treatment effect HRs (95% CI) for randomized controlled trials based on (1) PFS submodels or (2) PFS and symptom joint models

	OPTIMISMM		ELOQUENT-2		KarMMa-3		MM-003	
	PVd vs. Vd PFS HR (95% CI)		ERd vs. Rd PFS HR (95% CI)		Ide-cel vs. SoC PFS HR (95% CI)		POM + LoDex vs. HiDex PFS HR (95% CI)	
	PFS submodel	Joint model	PFS submodel	Joint model	PFS submodel	Joint model	PFS submodel	Joint model
QLQ-C30 pain	0.57 (0.45, 0.73)	0.55 (0.42, 0.71)	0.74 (0.62, 0.89)	0.72 (0.60, 0.87)	0.38 (0.29, 0.50)	0.37 (0.28, 0.49)	0.48 (0.38, 0.60)	0.44 (0.35, 0.56)
QLQ-MY20 disease symptoms	0.56 (0.44, 0.72)	0.53 (0.41, 0.69)	0.74 (0.61, 0.89)	0.72 (0.60, 0.87)	0.38 (0.28, 0.50)	0.36 (0.28, 0.48)	0.51 (0.41, 0.63)	0.49 (0.39, 0.62)
QLQ-C30 fatigue	0.55 (0.43, 0.71)	0.54 (0.41, 0.70)	0.75 (0.62, 0.90)	0.73 (0.61, 0.88)	0.38 (0.28, 0.50)	0.36 (0.27, 0.48)	0.48 (0.38, 0.60)	0.46 (0.36, 0.58)

Notes: Analyses specific to trial-specific HRQoL-evaluable populations and model covariates. See Tables 1 and 2 for details of the individual trials. *CI* confidence interval, *ERd* elotuzumab + lenalidomide + dexamethasone, *EORTC* European Organisation for Research and Treatment of Cancer, *HiDex* high-dose dexamethasone, *HR* hazard ratio, *HRQoL* health-related quality of life, *ide-cel* idecabtagene vicleucel, *Rd* lenalidomide + dexamethasone, *LoDex* low-dose dexamethasone, *PFS* progression-free survival, *POM* pomalidomide, *PVd* pomalidomide + bortezomib + dexamethasone, *QLQ-C30* EORTC Core Quality of Life questionnaire, *QLQ-MY20* EORTC Multiple Myeloma Module questionnaire, *SoC* standard of care, *Vd* bortezomib + dexamethasone

**Fig. 2:**
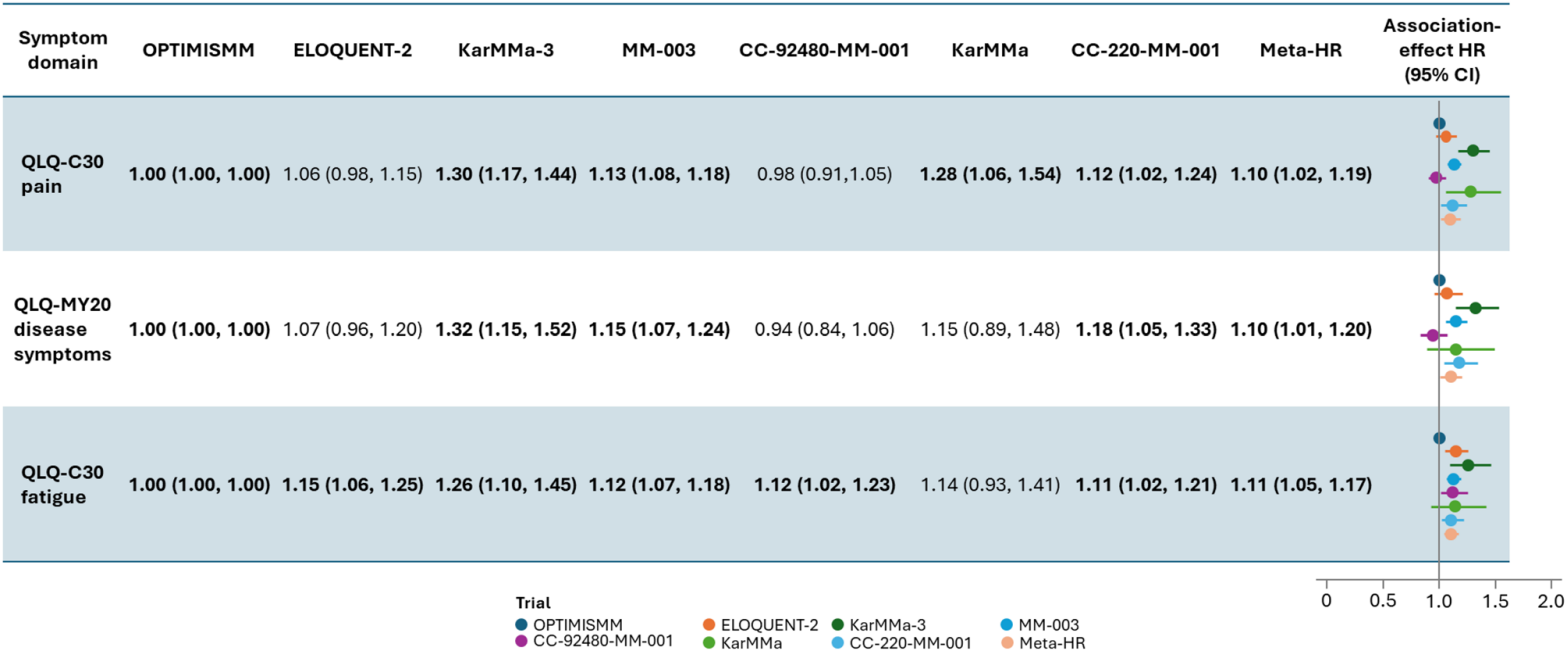
Association-effect HRs (trial-specific HRs and summary meta-HRs) characterizing the association between the rate of change in baseline scores for each symptom (pain, disease symptoms, and fatigue) and progression-free survival. Notes: See Tables 1 and 2 for details of the individual trials. Values represent HR (95% CI). Bolded values are statistically significant at the 0.05 significance level. A HR > 1 indicates that symptom worsening is associated with an increase in the hazard of a progression event (disease progression or death). Trials are ordered by increasing number of median prior lines. Analyses are specific to trial-specific HRQoL-evaluable populations and model covariates. The meta-HR represents a random-effects meta-analysis that synthesized the trial-specific association-effect HRs into one summary statistic for each symptom. *CI* confidence interval, *EORTC* European Organisation for Research and Treatment of Cancer, *HR* hazard ratio, *HRQoL* health-related quality of life, *QLQ-C30* EORTC Core Quality of Life questionnaire, *QLQ-MY20* EORTC Multiple Myeloma Module questionnaire

The trial-specific joint-model-association-effect HRs quantifying the association between each symptom of interest and PFS are presented in Fig. 2, with trials ordered by increasing number of median prior lines.

The QLQ-C30 and QLQ-MY20 domains both assessed pain, which was the most common symptom of concern among patients with RRMM identified in the literature review and verified by clinicians. Trial-specific joint-model-association-effect HRs (95% CI) for the QLQ-C30 pain models ranged from 0.98 (0.91, 1.05) for CC-92480-MM-001 to 1.30 (1.17, 1.44) for KarMMa-3. The joint-model-association-effect HRs were ≥ 1 and statistically significant for OPTIMISMM, KarMMa-3, MM-003, KarMMa, and CC-220-MM-001. For QLQ-MY20 disease symptoms, the joint-model-association-effect HRs (95% CI) ranged from 0.94 (0.84, 1.06) for CC-92480-MM-001 to 1.32 (1.15, 1.52) for KarMMa-3. Statistically significant results were observed for OPTIMISMM, KarMMa-3, MM-003, and CC-220-MM-001 models (all had HRs ≥ 1).

The trial-specific joint-model-association-effect HRs (95% CI) for the fatigue models ranged from 1.00 (1.00, 1.00) for OPTIMISMM to 1.26 (1.10, 1.45) for KarMMa-3. Statistically significant joint-model-association-effect HRs (all ≥ 1) were observed for the OPTIMISMM, ELOQUENT-2, KarMMa-3, MM-003, CC-92480-MM-001, and CC-220-MM-001 models.

Findings from sensitivity analyses assuming a current value association structure (based on a 10-point change from baseline) were similar to those from base case analyses assuming a current slope association structure (Supplementary Fig. S1). Findings were statistically significant for QLQ-C30 pain (OPTIMISMM, KarMMa-3, and MM-003), QLQ-MY20 disease symptoms (OPTIMISMM, KarMMa-3, MM-003, and CC-220-MM-001), and QLQ-C30 fatigue (OPTIMISMM, ELOQUENT-2, MM-003, and CC-220-MM-001).

### Meta-analysis of trial-specific joint models for PFS and symptoms

The meta-HRs (95% CI) synthesizing trial-specific joint models were 1.10 (1.02, 1.19) for QLQ-C30 pain and 1.09 (1.01, 1.20) for QLQ-MY20 disease symptoms, indicating that worsening in pain was associated with an increase in the hazard of disease progression or death across the trials. The I^2^ statistics for the QLQ-C30 pain and QLQ-MY20 disease symptoms meta-analyses were 92.1% and 85.6%, respectively. The QLQ-C30 fatigue meta-HR synthesizing the trial-specific joint models was 1.11 (95% CI: 1.05, 1.17; I^2^: 81.0%), indicating that worsening in fatigue was also associated with an increase in the hazard of disease progression or death across the trials.

## Discussion

This study demonstrated that deterioration in pain and fatigue was associated with an increased hazard of disease progression or death across numerous RRMM clinical trials. Statistically significant associations were observed in trial-specific models evaluating heterogeneous RRMM patient populations with varying extents of pretreatments, as well as in meta-analyses synthesizing the joint model outputs. Of note, the association held true for continuous treatments (immunomodulatory agents, proteasome inhibitors, and monoclonal antibodies alone or in combinations), as well as for single administration-based CAR T-cell therapy. This analysis adds to the literature by demonstrating a robust approach to (1) identifying and selecting patient-relevant symptoms based on a predefined literature review; (2) validating model selection with disease-specific clinical experts; and (3) using joint models to assess PFS and symptoms simultaneously.

Currently, there are mixed conclusions on the association between PFS and HRQoL in oncology research; however, differing methodological approaches have been used to assess this association. For example, no association was found in trial-level analyses of various cancer types using published aggregate data for PFS (e.g., published medians or HRs) and HRQoL (e.g., published scores) [9, 10]. In contrast, statistically significant associations between PFS and HRQoL have been noted in disease-specific (e.g., non-small cell lung cancer) analyses that used IPD from clinical trials [11, 31]. Similar to the current analysis, associations were observed in analyses that accounted for individual-level fluctuations in the outcomes of interest, rather than the overall average effects at the trial level. For both types of analyses, justification for the selected patient-reported symptom domains was either not reported or based solely on their common reporting in the published literature (without clinical consideration). Therefore, the transparent and robust approach from this current case could be applied and expanded to other disease areas.

Joint models have the strength of allowing for increased power as well as capturing correlation/uncertainty in the 2 outcomes of interest [13–15]. This was demonstrated in the current analysis, where treatment effects of PFS HRs from joint models of both PFS and symptoms had a reduction in uncertainty (i.e., narrower 95% CIs) compared with models based on PFS alone, independent of symptoms. The use of joint modeling in this analysis allowed for the simultaneous consideration of both PFS and disease progression-relevant symptoms, which captures information on both efficacy and patient experience. The use of joint models to analyze PFS and symptoms together should be considered as pre-specified analyses of clinical trials to support evaluations regarding the comparative effectiveness of new treatments.

Another strength of this analysis is the evaluation of the association of PFS and symptoms in numerous RRMM clinical trials, with a range of varying disease severity across the trial populations. Given this heterogeneity, trial-specific models were performed to assess the magnitude of the association across different target populations; however, the analysis was restricted to Celgene/BMS clinical trials with IPD available. Therefore, findings from this analysis may not be generalizable to broader clinical trials or beyond the RRMM setting and it will be important to assess these methods in the context of other trials in the future.

While the association-effect HR has the same definition across the trial-specific models, each HR is conditional on the inclusion of different covariates (based on trial-specific stratification factors and data availability). Since the included trial-specific analyses varied in terms of population characteristics and included covariates, random-effects meta-analysis models were used to summarize the association-effect HRs across the trials. As expected, the meta-analysis models had high heterogeneity, as indicated by the I^2^ statistics and these results should be interpreted within this context. Nonetheless, consistency in the direction of the association-effect HRs across the individual trials supports the robustness of the findings. Trends in the magnitude of the association-effect HRs from trial-specific joint models may provide clinical insights, such as an increasing magnitude in the association-effect HRs for trials with more pretreated populations. However, these patterns should not be overinterpreted without additional research using a meta-regression to synthesize the trial-specific adjusted HRs while accounting for additional between-study differences.

This analysis demonstrated a statistically significant association between PFS and symptoms, and future research can build upon this by exploring alternative clinically meaningful changes in symptoms to further explore the clinical significance of these findings. For example, studies could explore the concept of patient-relevant symptomatic disease progression as informed by the occurrence of clinically meaningful worsening in symptom scores (e.g., 10- or 15-point changes in symptom scores) in relation to PFS events. Future research would benefit from alignment with regulatory and/or HTA agencies on the most appropriate meaningful thresholds to consider in treatment benefit evaluations.

The timing and frequency of follow-up for patient-reported symptoms are important considerations and may constrain the feasibility of joint models. For example, minimal post-progression HRQoL evaluations (limited by data collected in clinical trials in patients after disease progression) make it challenging to fully assess the relationship of interest. This is a particular limitation for early-phase trials, such as CC-92480-MM-001 and CC-220-MM-001 from this current study, both of which had limited HRQoL follow-up and small sample sizes. Therefore, it would be of interest to update joint models for these trials once additional follow-up data become available. To address this limitation, clinical trials could be designed to better collect HRQoL data beyond disease progression.

Results from joint models can also be impacted by the frequency of HRQoL and tumor assessment evaluations for each trial, where longer time periods between evaluations may limit the ability to estimate the association between symptoms and disease progression due to recall bias, loss to follow-up, and/or missing HRQoL data. Additionally, the true date of disease progression and symptoms may not align with the recorded time based on the timing of assessments. Collectively, continued collection of HRQoL post disease progression would be beneficial when designing clinical trials, as this would allow for a more robust assessment of PFS and patient-reported symptom associations. The frequency of HRQoL assessments and availability of follow-up data pose limitations and should be considered when designing other disease-specific joint models of PFS patient-reported symptoms. Further, the joint model analyses were performed on the HRQoL-evaluable populations rather than the full intention-to-treat populations, which required patients to have completed at least one post-baseline HRQoL evaluation. While PFS curves from the intention-to-treat and HRQoL-evaluable populations were similar for each of the evaluated trials, there is a potential underlying selection bias for the analysis populations. In addition, the current analysis evaluated each symptom separately, but future work could explore the simultaneous analysis of multiple symptoms/cumulative effect of multiple worsening symptoms. Lastly, open-label design of the included trials may have introduced bias related to patients’ awareness of receiving treatment, which could influence symptom reporting, potentially leading to over- or underestimation of symptom severity.

One of the main assumptions of joint models is that the submodels are linked using an accurate association structure, as misspecification can introduce bias. Based on clinical input, the current slope association structure was selected, and additional sensitivity analyses were also conducted based on the current value association structure (assuming a 10-point change from baseline). Findings from sensitivity analyses were generally consistent with those from base case analyses.

## Conclusion

This study used IPD from numerous RRMM clinical trials to perform joint models of PFS and patient-relevant symptoms related to disease progression, concluding that worsening in pain and fatigue is associated with an increased hazard of disease progression or death. Overall, findings from this study suggest that PFS is associated with disease-relevant symptom worsening, highlighting the potential benefit of considering both PFS and symptoms when evaluating treatment benefits.

## Supplementary Information

Below is the link to the electronic supplementary material.

Supplementary Material 1

## Data Availability

The data that support the findings of this study are available from Bristol Myers Squibb but restrictions apply to the availability of these data, which were used under license for the current study, and so are not publicly available. Data are however available from the authors upon reasonable request and with permission of Bristol Myers Squibb.
