# Supplementary material for: Joint modeling of progression-free survival and patient-reported outcomes to evaluate the association between disease progression and symptoms among patients with relapsed/refractory multiple myeloma
Source: J Patient Rep Outcomes. 2025 Oct 14;9:118. doi: 10.1186/s41687-025-00943-9 (PMC12521705; doi:10.1186/s41687-025-00943-9)
Supplement: Supplementary file 1 — Supplementary Material 1 [file 41687_2025_943_MOESM1_ESM.docx]

**Table S1** Covariates selected for inclusion within the trial-specific models

| **Trial** | **Age, years** | **Number of prior regimens per year** | **Number of prior treatments** | **Time since diagnosis** | **High-risk cytogenetics** | **Prior agents or status** | **Beta-2-microglobulin, mg/L** | **Treatment** | **Baseline HRQoL score** |
| --- | --- | --- | --- | --- | --- | --- | --- | --- | --- |
| **OPTIMISMM** | ≤75 and >75^a^ | ***--*** | 1 vs >1^a^ | Continuous | ***--*** | ***--*** | <3.5 vs ≥3.5 to ≤5.5 vs >5.5^a^ | Study-specific | Continuous |
| **ELOQUENT-2** | ≤75 and >75 | ***--*** | 1 vs ≥2^a^ | Continuous | ***--*** | No vs prior thalidomide only vs other^a^ | <3.5 vs ≥3.5^a^ | Study-specific | Continuous |
| **KarMMa-3** | <65 vs ≥65^a^ | ***--*** | 2 vs 3–4^a^ | Continuous | High-risk vs normal-risk or unknown^a^ | ***--*** | ***--*** | Study-specific | Continuous |
| **MM-003** | ≤75 and >75^a^ | ***--*** | 2 vs ≥3^a^ | Continuous | ***--*** | Refractory vs relapsed and refractory vs bortezomib intolerant^a^ | ***--*** | Study-specific | Continuous |
| **CC-92480-MM-001** | ≤75 and >75 | ***--*** | Continuous | Continuous | ***--*** | ***--*** | ***--*** | ***--*** | Continuous |
| **KarMMa** | ≤75 and >75 | Continuous | ***--*** | ***--*** | ***--*** | ***--*** | ***--*** | ***--*** | Continuous |
| **CC-220-MM-001** | ≤75 and >75 | ***--*** | Continuous | Continuous | ***--*** | ***--*** | ***--*** | ***--*** | Continuous |

**Notes**: **^a^**Indicates stratification factor. See Tables 1 and 2 in the main article for details of the individual trials. *HRQoL* health-related quality of life

**Fig. S1** Trial- and symptom-specific joint-model-association-effect HRs (95% CI) from sensitivity analyses using current value (10-point change from baseline) as the joint-model-association structure


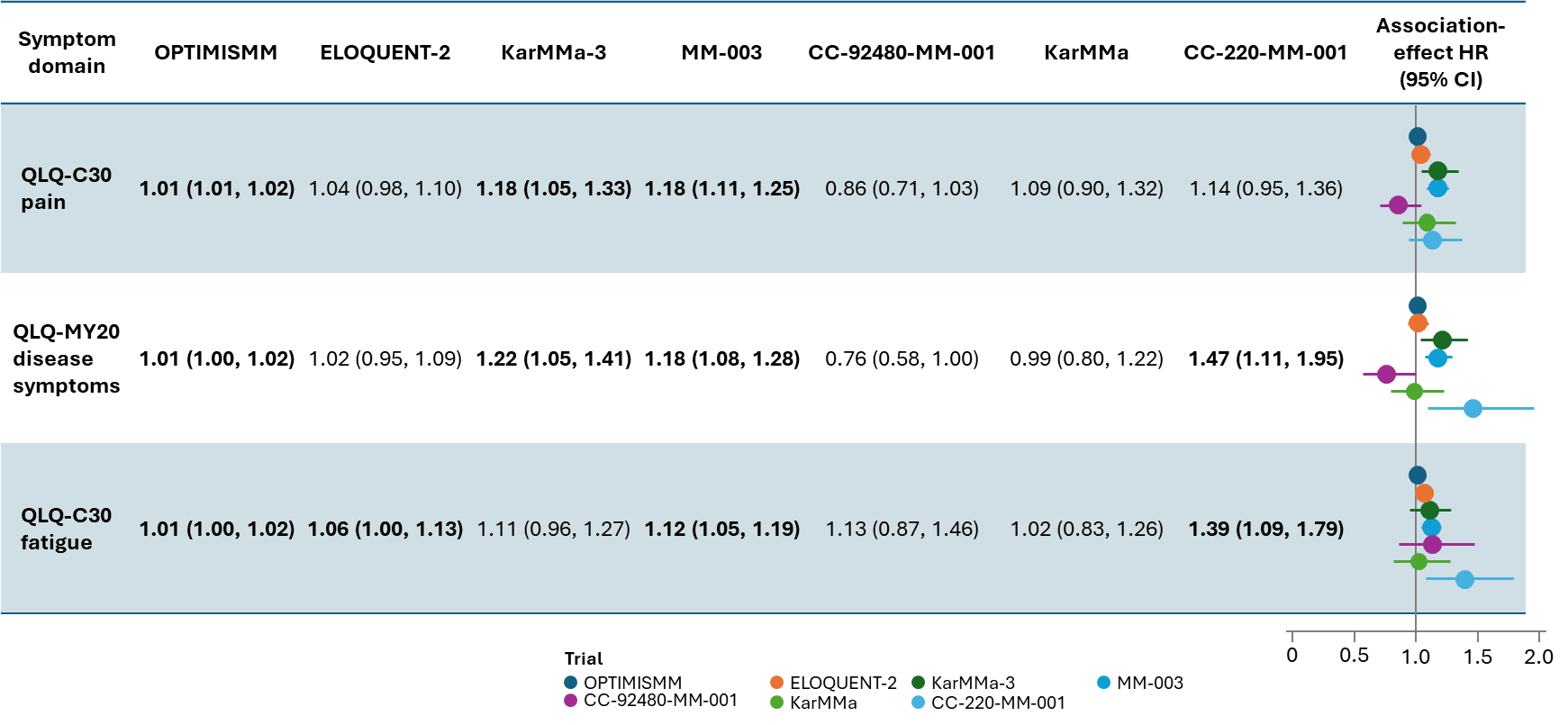


**Notes:** See Tables 1 and 2 in the main article for details of the individual trials. Bolded values are statistically significant at the 0.05 significance level. Trials are ordered by increasing number of median prior lines. Analyses are specific to trial-specific HRQoL-evaluable populations and model covariates. Based on a 10-point change from baseline score. *CI* confidence interval, *EORTC* European Organisation for Research and Treatment of Cancer, *HR* hazard ratio, *HRQoL* health-related quality of life, *QLQ-C30* EORTC Core Quality of Life questionnaire, *QLQ-MY20* EORTC Multiple Myeloma Module questionnaire.

**QLQ-C30 pain questions and scoring:**

During the past week:

- Question 9: Have you had pain?
  - Not at All = 1
  - A Little = 2
  - Quite a Bit = 3
  - Very Much = 4
- Question 19: Did pain interfere with you daily activities?
  - Not at All = 1
  - A Little = 2
  - Quite a Bit = 3
  - Very Much = 4

Raw Score = (Question 9 score + Question 19 score)/2

Pain Score = ((Raw Score−1)/3)*100

**QLQ-C30 fatigue questions and scoring:**

During the past week:

- Question 10: Did you need to rest?
  - Not at All = 1
  - A Little = 2
  - Quite a Bit = 3
  - Very Much = 4
- Question 12: Have you felt weak?
  - Not at All = 1
  - A Little = 2
  - Quite a Bit = 3
  - Very Much = 4
- Question 18: Were you tired?
  - Not at All = 1
  - A Little = 2
  - Quite a Bit = 3
  - Very Much = 4

Raw Score = (Question 10 score + Question 12 score + Question 18 score)/3

Fatigue Score = ((Raw Score−1)/3)*100

**QLQ-MY20 disease symptoms questions and scoring:**

During the past week:

- Question 31: Have you had bone aches or pain?
  - Not at All = 1
  - A Little = 2
  - Quite a Bit = 3
  - Very Much = 4
- Question 32: Have you had pain in your back?
  - Not at All = 1
  - A Little = 2
  - Quite a Bit = 3
  - Very Much = 4
- Question 33: Have you had pain in your hip?
  - Not at All = 1
  - A Little = 2
  - Quite a Bit = 3
  - Very Much = 4
- Question 34: Have you had pain in your arm or shoulder?
  - Not at All = 1
  - A Little = 2
  - Quite a Bit = 3
  - Very Much = 4
- Question 35: Have you had pain in your chest?
  - Not at All = 1
  - A Little = 2
  - Quite a Bit = 3
  - Very Much = 4
- Question 36: If you had pain did it increase with activity?
  - Not at All = 1
  - A Little = 2
  - Quite a Bit = 3
  - Very Much = 4

Raw Score = (Question 31 score + Question 32 score + Question 33 score + Question 34 score + Question 35 score + Question 36 score)/6

Disease Symptoms Score = ((Raw Score−1)/3)*100
